# Sar1 GTPase Activity Is Regulated by Membrane Curvature*[Fn FN3]^♦^

**DOI:** 10.1074/jbc.M115.672287

**Published:** 2015-11-06

**Authors:** Michael G. Hanna, Ioanna Mela, Lei Wang, Robert M. Henderson, Edwin R. Chapman, J. Michael Edwardson, Anjon Audhya

**Affiliations:** From the ‡ Department of Biomolecular Chemistry, University of Wisconsin-Madison School of Medicine and Public Health, Madison, Wisconsin 53706,; the § Department of Pharmacology, University of Cambridge, Tennis Court Road, CB2 1PD Cambridge, United Kingdom, and; the ¶ Department of Neuroscience, University of Wisconsin-Madison School of Medicine and Public Health, Madison, Wisconsin 53705

**Keywords:** COPII, endoplasmic reticulum (ER), GTPase, membrane bilayer, membrane transport

## Abstract

The majority of biosynthetic secretory proteins initiate their journey through the endomembrane system from specific subdomains of the endoplasmic reticulum. At these locations, coated transport carriers are generated, with the Sar1 GTPase playing a critical role in membrane bending, recruitment of coat components, and nascent vesicle formation. How these events are appropriately coordinated remains poorly understood. Here, we demonstrate that Sar1 acts as the curvature-sensing component of the COPII coat complex and highlight the ability of Sar1 to bind more avidly to membranes of high curvature. Additionally, using an atomic force microscopy-based approach, we further show that the intrinsic GTPase activity of Sar1 is necessary for remodeling lipid bilayers. Consistent with this idea, Sar1-mediated membrane remodeling is dramatically accelerated in the presence of its guanine nucleotide-activating protein (GAP), Sec23-Sec24, and blocked upon addition of guanosine-5′-[(β,γ)-imido]triphosphate, a poorly hydrolysable analog of GTP. Our results also indicate that Sar1 GTPase activity is stimulated by membranes that exhibit elevated curvature, potentially enabling Sar1 membrane scission activity to be spatially restricted to highly bent membranes that are characteristic of a bud neck. Taken together, our data support a stepwise model in which the amino-terminal amphipathic helix of GTP-bound Sar1 stably penetrates the endoplasmic reticulum membrane, promoting local membrane deformation. As membrane bending increases, Sar1 membrane binding is elevated, ultimately culminating in GTP hydrolysis, which may destabilize the bilayer sufficiently to facilitate membrane fission.

## Introduction

Approximately one-third of all translated proteins in mammalian cells are predicted to enter the secretory pathway (1). These diverse cargoes must be folded properly within the oxidative environment of the endoplasmic reticulum (ER)^4^ lumen and subsequently packaged into coated vesicles or tubules for export. Components of the cytosolic coat protein II (COPII) complex play key roles in ER membrane remodeling and scission to bud transport carriers (1–4). In particular, the conserved Sar1 GTPase has been implicated in multiple steps of this process. Current models suggest that in the presence of its guanine nucleotide exchange factor (GEF) Sec12, which is enriched at subdomains of the ER that synthesize COPII-coated carriers, GTP-bound Sar1 exposes an amphipathic helix that penetrates the outer leaflet of the membrane (5–9). This type of membrane penetration induces positive curvature that is further stabilized when Sar1-GTP is bound by its major effector, the Sec23–24 heterodimer, which exhibits a concave surface (10, 11). Additionally, recruited Sec23–24 complexes generate an adaptor layer for Sec13–31 lattice assembly, completing the COPII vesicle coat (12). Recent studies have highlighted the flexibility exhibited by the inner and outer COPII layers, enabling the formation of small vesicles (∼50–100 nm in diameter) and elongated tubules (∼300–400 nm in length), which can capture and traffic both small and large cargoes, respectively, away from the ER (13, 14). However, mechanisms governing the location and timing of carrier scission have not been clearly defined. In particular, how GTP hydrolysis on Sar1 contributes to this process continues to be debated.

In cell-free assays conducted more than 2 decades ago, poorly hydrolysable analogs of GTP were shown to be sufficient to promote COPII-mediated vesicle formation and release, suggesting that GTP hydrolysis was dispensable for the budding reaction (1). Similar studies were conducted more recently and confirmed this finding (14). However, in both cases, Sar1-dependent vesicle release was measured following differential centrifugation and/or prolonged periods of incubation with COPII components and nucleotide analogs, which may have led to the unintentional detachment of nascent transport carriers from ER membranes. Using synthetic giant unilamellar vesicles (GUVs), another report showed that Sar1 alone was capable of deforming membranes and generating small vesicles independently of GTP hydrolysis (15). However, a high concentration of Sar1 (>7 μm) was necessary for this effect, which may not be physiologically relevant in the context of COPII-mediated budding *in vivo*. Moreover, the electron microscopy (EM)-based methods employed in this study required mechanical perturbations, including fixation and dehydration in negative staining preparations and liquid blotting in cryogenic approaches, which can perturb samples sufficiently to induce artifacts (16). In contrast to these studies, others have shown that inhibition of GTP hydrolysis on Sar1 strongly inhibits vesicle budding from ER microsomes or vesiculation of synthetic GUVs (7, 8, 13, 16–18), raising the possibility that nucleotide hydrolysis contributes to the membrane fission reaction necessary to detach transport carriers. Although difficult to reconcile, these conflicting reports highlight the need for minimally invasive techniques to study the role of Sar1 in membrane remodeling, while still maintaining sufficiently high spatial and temporal resolution to interrogate the process.

Another consistent observation from these studies indicates that Sar1 is capable of associating with lipid bilayers that exhibit a range of curvatures, in a manner dependent on the amino-terminal amphipathic helix of Sar1. Specifically, Sar1 has been shown to bind to both highly curved liposomes (nanometers in diameter) and flat GUVs (microns in diameter) (7, 15). However, Sar1 remodels GUVs over relatively long time scales (minutes to hours), generating heterogeneous tubules that range in diameter from ∼40 to 250 nm (15). Depending on the size of the tubule, Sar1 can form structurally unique scaffolds, varying from ordered lattices on membranes possessing shallow curvature to fragmented arrays on bilayers of higher curvature (15). The difference in its membrane binding properties may be related to its dual role in the formation and budding of COPII-coated transport carriers. However, apart from structural studies and largely qualitative measurements of bilayer association, the biophysical characteristics of Sar1 membrane binding have not been clearly defined. Additionally, it remains unknown how membrane curvature impacts GTP hydrolysis on Sar1. Here, we investigate these properties using Sar1 isoforms isolated from three distinct species. Our data demonstrate that membrane curvature strongly influences both the affinity of Sar1 for membranes and its rate of GTP hydrolysis in an evolutionarily conserved manner. Collectively, our data are most consistent with a model in which Sar1 promotes membrane scission at bud necks in a curvature-dependent process that involves GTP hydrolysis.

## Experimental Procedures

### 

#### 

##### Caenorhabditis elegans Growth, Maintenance, RNA Interference, and Live Imaging

All *C. elegans* strains used in this study were derived from the Bristol strain N2, which was described previously (19, 20). Double-stranded RNA (dsRNA) was synthesized from templates prepared by PCR to amplify *C. elegans* genomic DNA. For RNAi experiments, early L4 stage hermaphrodites were soaked in dsRNA for 24 h at 20 °C within a humidified chamber. Animals were then allowed to recover for 24–48 h before analyzing them for embryo production or mounting onto a 10% agarose pad in a 4-μl suspension of polystyrene beads to immobilize them for imaging (21). Images were acquired on a swept-field confocal microscope (Nikon Ti-E), using a Nikon ×60, 1.4 numerical aperture Planapo oil objective lens and a Roper CoolSnap HQ2 CCD camera. Acquisition parameters were controlled by Nikon Elements software, and image analysis was conducted using Metamorph software.

##### Recombinant Protein Expression, Purification, and Mass Determination

All *C. elegans* COPII components were amplified from a *C. elegans* cDNA library, and Sanger sequencing was used to confirm their identity. Proteins were expressed as His_6_-SUMO fusions and purified using nickel-nitrilotriacetic acid-agarose resin in Sar1 buffer (25 mm HEPES, pH 7.2, 100 mm NaCl, and 1 mm MgCl_2_). Sumo protease was used to remove the His_6_-Sumo tag, and the cleaved proteins were subjected to size-exclusion chromatography, which was coupled to a Wyatt mini-DAWN TREOS three-angle light scattering detector and a Wyatt Optilab T-rEX refractive index detector. Data were collected at a flow rate of 0.5 ml/min and analyzed using ASTRA software to determine molecular mass (22). For *C. elegans* SAR-1, protein purification was carried out in the presence of either GDP or GTP (500 μm each). Specifically, a 30-fold molar excess of nucleotide was present during Sumo protease cleavage (16 h at 4 °C) to ensure complete incorporation onto SAR-1, as described previously (11). Furthermore, nucleotide-bound SAR-1 was additionally gel-filtered in the presence of the appropriate nucleotide (500 μm) prior to use in all assays.*C. elegans* SEC-23/SEC-24.2 and *Saccharomyces cerevisiae* Sec23p-Sec24p complexes were purified similarly, with the exception of the buffer used (25 mm HEPES, pH 7.2, 160 mm KOAc, and 1 mm MgCl_2_). Human and yeast forms of Sar1 were purified as described previously (1).

##### Synthetic Liposome Generation and Size Determination

Liposomes were generated as described previously (13). Phospholipids in chloroform were mixed to generate lipid mixtures, including the “major/minor mix” (lipid, mol %) as follows: 1,2-dioleoyl-*sn*-glycero-3-phosphocholine (DOPC), 50;1,2-dioleoyl-*sn*-glycero-3-phosphoethanolamine (DOPE), 21; 1,2-dioleoyl-*sn*-glycero-3-phosphoserine (DOPS), 8; 1,2-dioleoyl-*sn*-glycero-3-phosphate (DOPA), 5; phosphatidylinositol (PI), 9; phosphatidylinositol 4-phosphate (PI4P), 2.2; phosphatidylinositol 4,5-bisphosphate (PI(4,5)P_2_), 0.8; diacylglycerol (DAG), 2; 1,2-dioleoyl-*sn*-glycero-3-phosphoethanolamine-*N*-(7-nitro-2–1,3-benzoxadiazol-4-yl), 2, and supplemented with 20% cholesterol, which was shown previously to facilitate maximal binding of Sar1 isoforms (13). When *S. cerevisiae* proteins were utilized, 20% ergosterol was used in place of cholesterol. Lipids were dried, resuspended in Sar1 buffer, and subjected to extrusion through a nitrocellulose filter (Whatman) of a desired pore size. Liposomes were analyzed by dynamic light scattering using a Wyatt DynaPro NanoStar to determine their average diameters.

##### Stopped-flow Rapid Kinetic Analysis and Co-sedimentation Analysis

Stopped-flow rapid kinetic analysis and co-sedimentation experiments were carried out as described previously (23). Briefly, SAR-1 (8 μm) was incubated with GTP (100 μm) in Sar1 buffer before being injected into the stopped-flow chamber. An equal volume of Sar1 buffer containing a desired liposome dilution was simultaneously injected to initiate SAR-1 binding. Liposomes contained the following phospholipid composition (lipid, mol %): DOPC, 71; DOPE, 15; DOPS, 8; DOPA, 5; dansyl-PE, 1, and were supplemented with 20% cholesterol (major/minor mix lacking phosphoinositides and DAG). For co-sedimentation analysis, SAR-1 (4 μm) was incubated with 1 mm GMP-PNP in Sar1 buffer and supplemented with a desired liposome concentration. Liposomes were composed of 66% DOPC, 21% DOPE, 8% DOPS, 5% DOPA, and supplemented with 20% cholesterol. Samples were mixed in a TLA100 tube for 15 min at room temperature and then centrifuged at 100,000 × *g* for 45 min. The supernatant was collected, mixed with sample buffer, and resolved on an SDS-polyacrylamide gel for densitometry to determine SAR-1 depletion.

##### Sar1 GTPase Activity Assays

The GTPase/GAP/GEF-Glo system (Promega) was used to measure the concentration of GTP remaining after incubation with Sar1 isoforms (24). Sar1 (3.1 μm, unless otherwise noted) was added to the reaction before the addition of GTP (5 μm) in a 25-μl reaction (Corning Costar 3912 solid white 96-well plate) in the supplied GEF buffer containing MgCl_2_. The addition of GTP initiated the reaction, which was conducted at 25 °C. After 2 h, an equal volume of the GTPase-Glo reagent was added to stop the reaction, incubated for 30 min, and followed by the addition of an equal volume of the detection reagent for 10 min. Luminescence was measured on a 96-well plate reader. All reactions were performed in triplicate and repeated independently on three separate occasions. Reactions with *S. cerevisiae* proteins were performed similarly, except that the initial reactions were carried out at 30 °C. Malachite Green GTPase assays were conducted by first incubating SAR-1^GDP^ with liposomes (1.27 mm) of a specific diameter for 10 min at room temperature. The GTPase reaction was initiated with the addition of GTP (300 μm) to a final reaction volume of 20 μl. In some cases, recombinant Sec23-Sec24 (10 μm, unless otherwise noted) was also present in the reaction prior to GTP addition. The reaction was allowed to continue for 1 h at room temperature. Malachite Green reagent (w/v %: Malachite Green, 0.027; poly(vinyl alcohol), 0.773; ammonium molybdate, 1.01) was added to the reaction to a final volume of 440 μl and allowed to incubate at room temperature for 2 h for the reaction with free inorganic phosphate to proceed to completion (indicated by a colorimetric change). After incubation with the Malachite Green reagent, the reaction was quenched with a sodium citrate solution (w/v %: 34; 50 μl) and incubated at room temperature for 5 min before absorbance measurements were taken (636 nm).

##### Fluorescence Quenching Assays

SAR1B^W86F,W192F^ (SAR1B Trp reporter) was engineered using site-directed mutagenesis and purified similarly to the wild type protein. Acrylamide quenching measurements were carried out as described previously (25). Briefly, a 2.0 m stock of acrylamide was dissolved in Sar1 buffer (with the addition of 1 mm DTT). The aqueous acrylamide quencher was added to the SAR1B-Trp reporter (1 μm) and supplemented with either 1 mm GDP or 1 mm GTP in the presence or absence of 2 mm lipids (mol %: DOPC, 75; DOPE, 12; DOPS, 8; DOPA, 5) as indicated. Emission spectra were subtracted for blank, lipids, nucleotide, and acrylamide and integrated from 300 to 400 nm to calculate fluorescence intensity. The fluorescence intensity was plotted *versus* quencher concentration (0–140 mm), and the degree of quenching was analyzed using the Stern-Volmer equation (Equation 1),

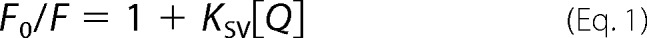
 where *F_o_* and *F* are the fluorescence intensities in the absence and presence of acrylamide, respectively; *K*_SV_ is the Stern-Volmer constant for collisional quenching, and [*Q*] is the concentration of aqueous quencher.

To measure the depth of penetration of the SAR1B Trp reporter into the membrane bilayer, parallax analysis was performed (25). Briefly, the fluorescence of the SAR1B-Trp reporter was individually measured in the presence of two separate membrane-embedded quenchers of distinct depths. The distance of the single Trp reporter from the bilayer center (*Z*_CF_) was determined using parallax analysis as given by Equation 2,

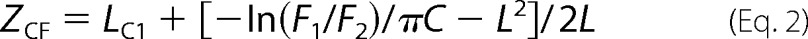
 where *L*_C1_ represents the distance from the bilayer center to the shallow quencher; *C* is the mole fraction of the quencher divided by the lipid area; *F*_1_ and *F*_2_ are the relative fluorescence intensities of the shallow (6,7-Br_2_-PC) and deep quenchers (11,12-Br_2_-PC), respectively, and *L* is the difference in depth between the two quenchers. Brominated lipid concentrations were varied by substituting DOPC.

##### Atomic Force Microscopy

AFM imaging was performed using a Bruker Dimension FastScan instrument. All imaging was conducted under fluid using FastScan D cantilevers (Bruker). Their resonant frequencies under fluid were 110–140 kHz, and the actual scanning frequencies were ∼5% below the maximal resonance peak. Lipid mixtures containing either a “basic mix” of lipids (70% DOPC, 15% DOPE, and 15% DOPS) or the major/minor mix (52% DOPC, 21% DOPE, 8% DOPS, 5% DOPA, 9% PI, 2.2% PI4P, 0.8% PI(4,5)P_2_, 2% DAG, and supplemented with 20% cholesterol) were dried under nitrogen and hydrated in Biotechnology Performance Certified water overnight. Suspensions were probe-sonicated at an amplitude of 10 μA until the mixture became transparent. Liposomes were incubated in the presence or absence of proteins for 30 min and then placed on freshly cleaved mica. In each case, 40 μl of the liposome mixture and an equal volume of buffer (50 mm HEPES, pH 7.6, 100 mm NaCl, and 1 mm MgCl_2_) were applied to the mica surface. The mica was washed three times with the same buffer and placed in the fluid cell of the atomic force microscope. The assembled lipid bilayer was immersed in 150 μl of buffer, and all imaging was performed at room temperature. For the buffer-exchange experiments, buffer containing 1 mm GTP, GMP-PNP, or GDP was introduced into the fluid cell of the microscope. AFM images were acquired at a rate of four frames/min and plane-fitted to remove tilt. Each scan line was fitted to a first-order equation.

## Results

### 

#### 

##### C. elegans Sar1 Regulates the Secretion of ER-derived Transport Carriers to Maintain Germ Line Tissue Architecture

**FIGURE 1.**
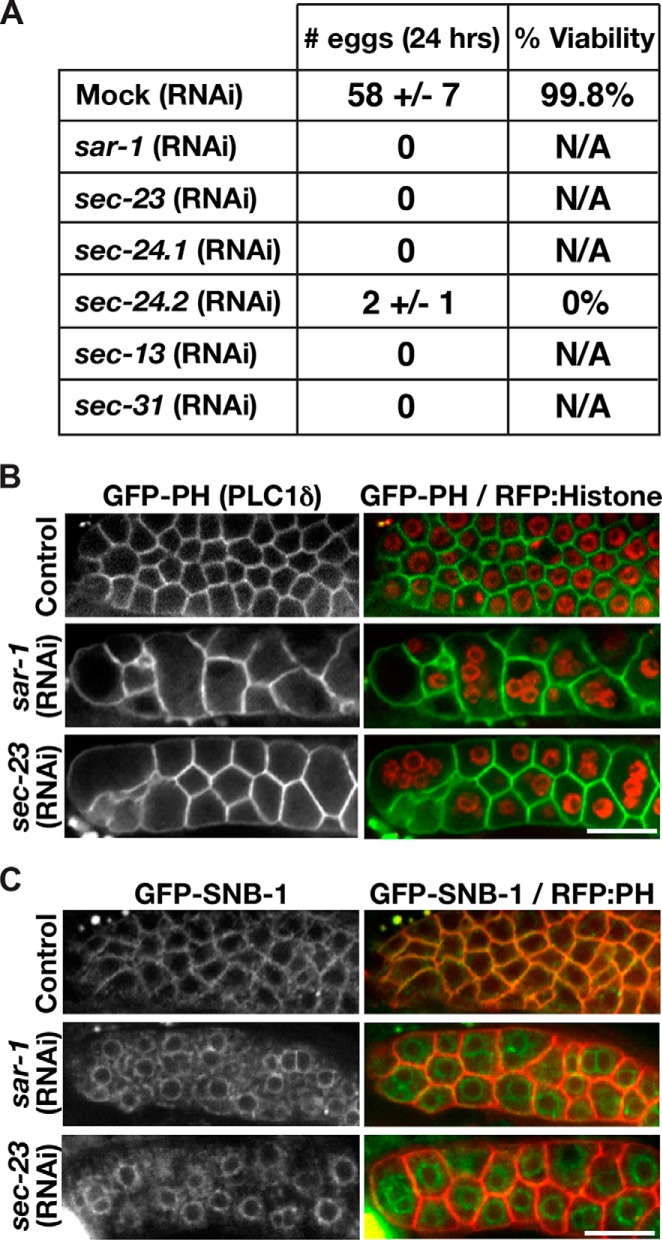
***C. elegans* SAR-1 is functionally conserved.** *A,* effect of depleting each of the *C. elegans* COPII subunits was assessed by measuring the ability of treated animals to produce embryos during a 24-h period following exposure to various dsRNAs. Embryos produced following depletion of SEC-24.2 were osmotically sensitive and did not remain viable beyond the one-cell stage. *N/A*, not applicable. *B,* transgenic animals co-expressing a GFP fusion to the pleckstrin homology (*PH*) domain of PLC1δ, which binds to phosphatidylinositol 4,5-bisphosphate present on the surface of membrane compartments throughout the germ line, and a red fluorescent protein (*RFP*) fusion to histone H2B (HIS-58) were treated with dsRNAs targeting COPII subunits for 24 h, and the distal portions of their germ lines were imaged 24 or 48 h later (for SEC-23 and SAR-1, respectively) using swept field confocal optics. *Scale bar,* 5 μm. *C,* transgenic animals co-expressing a GFP fusion to SNB-1, an integral membrane protein produced in the ER and trafficked to the surface of compartments throughout the germ line, and a red fluorescent protein fusion to the PLC1δ pleckstrin homology domain, were treated as described in *B. Scale bar,* 5 μm.

The Sar1 GTPase plays a conserved role in protein and lipid secretion in several organisms, including yeast, *Drosophila*, and mammals (1, 26, 27). In humans, several distinct mutations affecting one of the Sar1 isoforms (SAR1B) have been implicated in chylomicron retention disease (Anderson disease), an early onset, inherited lipid malabsorption disorder characterized by hypocholesterolemia (28, 29). However, the contribution of Sar1 to other developmental processes, including tissue morphogenesis, remains largely uncharacterized. To investigate a potential role for Sar1 GTPases in this process and to verify that *C. elegans* SAR-1 regulates protein secretion from the ER, we conducted a series of depletion experiments using the *C. elegans* germ line as a model system. An important feature of the *C. elegans* reproductive system is its amenability to RNA interference (RNAi)-mediated depletion (30). Consistent with previous work, inhibition of the single Sar1 isoform expressed in worms (ZK180.4) results in potent sterility (Fig. 1*A*) (31). Further examination of germ line tissue depleted of SAR-1 revealed a dramatic defect in the organization of membranes that normally partition individual nuclei into compartments within the syncytial stem cell niche (Fig. 1*B*). A nearly identical phenotype was observed upon depletion of the COPII subunit SEC-23, suggesting that the defect was a consequence of inhibiting COPII-mediated secretion (Fig. 1*B*). Consistent with this idea, trafficking of integral membrane secretory cargoes, including the secretory vesicle-associated SNARE synaptobrevin (SNB-1), from the ER was potently blocked following depletion of SAR-1 or SEC-23 (Fig. 1*C*). These data highlight a conserved function for *C. elegans* SAR-1 and COPII in protein secretion and further demonstrate a key role for COPII-mediated vesicle transport during tissue morphogenesis.

##### Stable Membrane Penetration of Sar1 Is Dependent upon an Association with GTP

**FIGURE 2.**
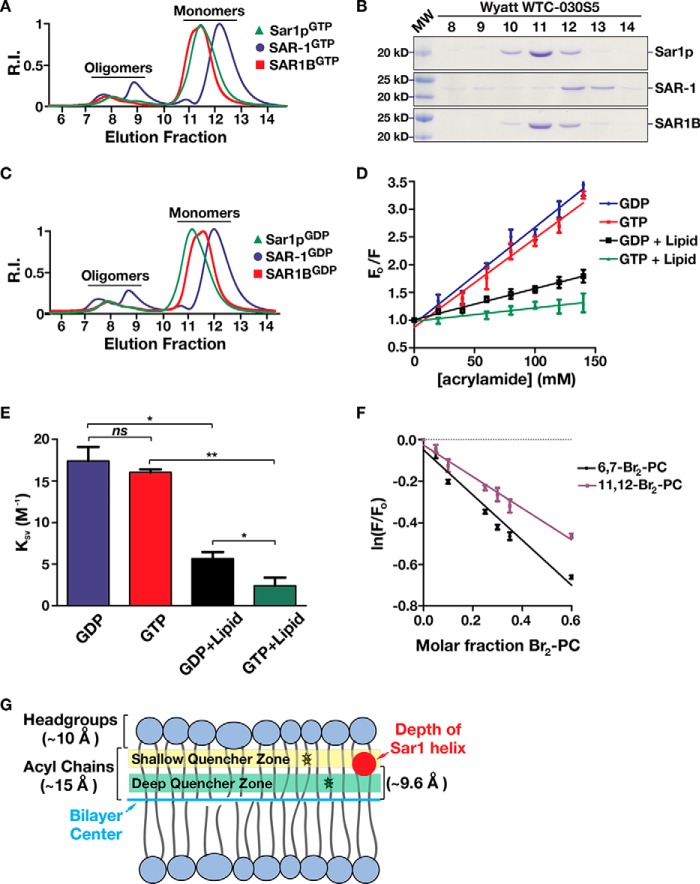
**GTP facilitates stable Sar1 membrane penetration.** *A,* refractometer traces from multiangle light scattering measurements of Sar1^GTP^ isoforms from various species are shown. Samples were applied onto a size-exclusion chromatography column equilibrated in 500 μm GTP before light-scattering measurements were taken. The relative intensities (*R.I.*) of fractions eluted are shown following normalization. *B,* samples (Sar1^GTP^ isoforms) eluted from a Wyatt WTC-030S5 size-exclusion column subsequent to multiangle light scattering analysis were separated by SDS-PAGE analysis and stained with Coomassie. *C,* refractometer traces from multiangle light scattering measurements of Sar1^GDP^ isoforms from various species are shown. Samples were applied onto a size-exclusion chromatography column equilibrated in 500 μm GDP before light-scattering measurements were taken. The relative intensity values of fractions eluted are shown following normalization. *D,* various concentrations of aqueous acrylamide were added to SAR1B^W86F,W192F^ (1 μm) in the presence of either GDP alone, GTP alone, GDP with liposomes, or GTP with liposomes. Liposomes were composed of 66% DOPC, 21% DOPE, 8% DOPS, 5% DOPA and supplemented with 20% cholesterol. Tryptophan fluorescence emission spectra were recorded from 300 to 400 nm and integrated to determine fluorescence intensity. *E,* Stern-Volmer constant (*K*_SV_) calculations reveal that the amphipathic tail of SAR1B is solvent-exposed when the GTPase is bound to either GDP or GTP. In the presence of liposomes, the amphipathic tail of SAR1B^GTP^ is embedded more stably in the membrane bilayer and protected from the aqueous quencher, as compared with SAR1B^GDP^. The statistical significance of pairwise differences was calculated using a *t* test. *ns,* not significant; *, *p* < 0.05; **, *p* < 0.01. *F,* SAR1B^GTP^ was added to liposomes harboring an embedded membrane quencher (shallow: 6,7-Br_2_-PC; deep: 11,12-Br_2_-PC) at the indicated mol %. Tryptophan fluorescence emission spectra were recorded from 300 to 400 nm and integrated to determine fluorescence intensity. Only the 60 mol % liposome condition was used for parallax calculation analysis, which revealed that the SAR1B amphipathic tail penetrates to ∼9.6 Å from the bilayer center. *G,* schematic illustrating the penetration depth of the SAR1B amphipathic helix relative to the positions of a shallow membrane quencher (6,7-Br_2_-PC), a deep membrane quencher (11,12-Br_2_-PC), and the center of the bilayer. Approximate sizes of the lipid headgroups and acyl chains are indicated.

Because our findings indicated that *C. elegans* SAR-1 is functionally conserved, we decided to purify a recombinant form of the GTPase and analyze its membrane binding capabilities *in vitro*. For these experiments, nucleotide was loaded onto SAR-1 by purifying the protein in the presence of a large molar excess of GTP or GDP (500 μm), as described previously (11). Because previous work indicates that Sar1 GTPases exhibit minimal intrinsic activity in the absence of a guanine nucleotide-activating protein (GAP), we decided to use GTP in these experiments as opposed to a poorly hydrolysable analog (32). Size-exclusion chromatography and multiangle light scattering conducted in the presence of nucleotide (500 μm GTP or GDP) indicated that recombinant SAR-1 loaded with either GTP or GDP formed mainly monodisperse monomers in solution, with small populations of dimers and larger oligomers (Fig. 2, *A–C,* and Table 1). Similar results were obtained using recombinant forms of yeast Sar1p and human SAR1B (Fig. 2, *A–C*, and Table 1). These data indicate that Sar1 isoforms exist largely as monomers in solution, but they are also capable of self-association.

**TABLE 1:** **Molecular masses of monomeric Sar1 isoforms determined experimentally using size-exclusion chromatography-multiangle light scattering**

Sar1 isoform	Predicted molecular mass (kDa) based on amino acid content	Experimentally determined mass (kDa) when bound to GDP	Experimentally determined mass (kDa) when bound to GTP
*S. cerevisiae* Sar1p	21.5	20.3 ± 0.5	20.7 ± 0.4
*C. elegans* SAR-1	21.7	21.6 ± 0.4	21.5 ± 0.4
*Homo sapiens* SAR1B	22.4	16.8 ± 0.5	19.3 ± 0.4

We next investigated the manner by which Sar1 binds to lipid bilayers. Although the amphipathic amino terminus of Sar1 is known to play an essential role in membrane association and tubulation, relatively little is known about its dynamic properties. To address this issue, we used a technique that measures tryptophan fluorescence quenching by aqueous acrylamide, both in the presence and absence of membranes (33). For these experiments, we used a form of human SAR1B, which harbors only a single naturally occurring tryptophan reporter at position 7 within the amino-terminal amphipathic helix (SAR1B^W86F,W192F^), enabling us to examine its accessibility. The protein was purified in the presence of GDP and subsequently incubated with a 100-fold molar excess of GTP for 30 min at room temperature, which was shown previously to be sufficient for nucleotide exchange (7). This form of SAR1B (1 μm) exhibited efficient quenching of fluorescence upon the addition of acrylamide, suggesting that the amphipathic helix was exposed in solution (Fig. 2, *D* and *E*, and Table 2). We also measured changes in tryptophan fluorescence when GDP-bound SAR1B^W86F,W192F^ was incubated with increasing concentrations of acrylamide. In contrast to the idea that the amphipathic helix is buried within the core of Sar1 when bound to GDP, we found that the addition of acrylamide resulted in equally efficient quenching with nearly identical kinetics to the GTP-bound protein (Fig. 2, *D* and *E*, and Table 2). These data argue that the amphipathic helix of Sar1 is solvent-exposed irrespective of its nucleotide-bound state.

**TABLE 2:** **Stern-Volmer constants determined for the SAR1B amphipathic helix under various conditions**

Condition	*K*_SV_
	*m*^−*1*^
GDP (in solution)	17.2 ± 1.0
GTP (in solution)	16.0 ± 0.9
GDP + liposomes	6.5 ± 0.5
GTP + liposomes	2.4 ± 0.5

When examined in the presence of membranes, we found that the GTP-bound form of SAR1B was highly resistant to tryptophan fluorescence quenching, suggesting that the amphipathic helix becomes buried within the hydrophobic core of the lipid bilayer under these conditions and is protected from acrylamide in solution (Fig. 2, *D* and *E,* and Table 2). Surprisingly, GDP-bound SAR1B also exhibited reduced fluorescence quenching when membranes were present, although the effect was not as pronounced as compared with GTP-bound SAR1B (Fig. 2, *D* and *E*, and Table 2). These data are consistent with the idea that SAR1B can associate with membranes, irrespective of the bound nucleotide. However, GTP-binding appears to promote more stable membrane penetration of the SAR1B amphipathic helix, which likely supports membrane bending during the formation of COPII-coated transport carriers.

To determine the extent to which the SAR1B amino-terminal amphipathic helix inserts into lipid bilayers, we measured tryptophan fluorescence quenching mediated by brominated phospholipids that are modified at specific positions along their acyl chains (34). Parallax analysis demonstrated that the single tryptophan reporter in the helix penetrates ∼5 Å into the hydrophobic core of the outer leaflet, just below the hydrophilic headgroups, in the presence of GTP (Fig. 2, *F* and *G*; see “Experimental Procedures” for calculations). These data provide a mechanistic basis for the ability of Sar1 isoforms to bind and bend membranes.

##### Sar1 Is the Curvature-sensing Component of the COPII Complex

**FIGURE 3.**
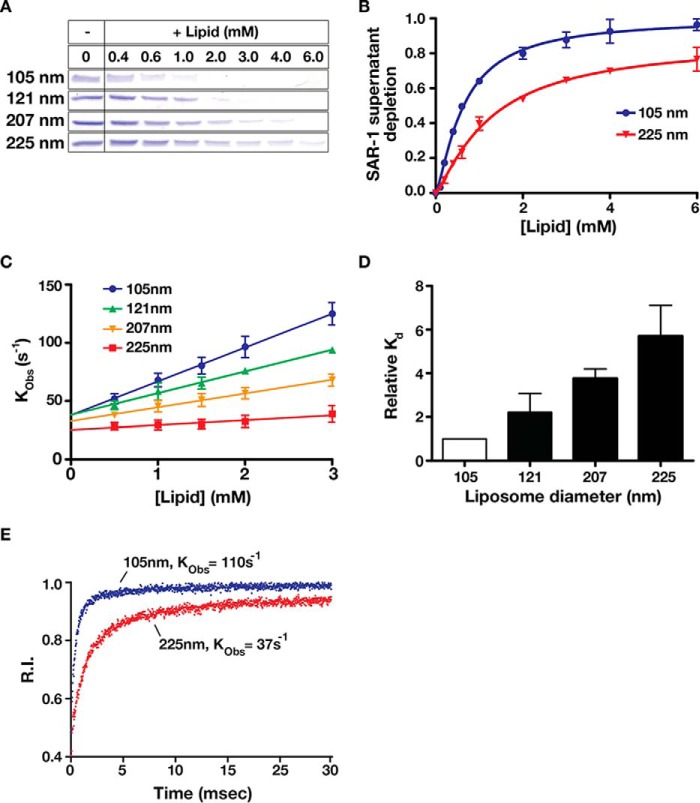
**Sar1 preferentially binds to membranes that exhibit elevated curvature.** *A,* SAR-1^GMP-PNP^ (4 μm) was incubated in the presence of a range of liposome concentrations of varying diameter (105–225 nm) before being subjected to ultracentrifugation. Supernatants were collected after centrifugation, resolved by SDS-PAGE, and stained using Coomassie. *B,* densitometry measurements were used to determine relative depletion of SAR-1 from the supernatant, and these values were plotted against liposome concentration. *C,* SAR-1^GTP^ (8 μm) was rapidly mixed with an equal volume of liposomes (composed of 71% DOPC, 15% DOPE, 8% DOPS, 5% DOPA, 1% dansyl-PE, and supplemented with 20% cholesterol) of varying diameters at the indicated concentrations using stopped-flow rapid kinetic analysis. Liposomes harboring the membrane-embedded dansyl moiety were used to measure SAR-1 fluorescence resonance energy transfer after solutions were mixed. *K*_Obs_ for each condition was plotted against liposome concentration. *D,* dissociation constants (*K_d_*) were measured for each liposome diameter described in *C* and divided by the calculated *K_d_* of the 105 nm condition to determine the relative changes in apparent affinity between liposome conditions. As liposome diameter increases, *K_d_* increases indicating lower binding affinities. *E,* representative stopped-flow data recorded upon mixing SAR-1 (8 μm) with liposomes (3 mm) of two different sizes. Fluorescence resonance energy transfer between the tryptophan residues in the SAR-1 amphipathic helix and membrane-embedded dansyl was measured over 30 ms.

The formation of all transport carriers requires stepwise changes in local membrane curvature that ultimately result in membrane scission. We hypothesized that a curvature-sensing mechanism may exist to facilitate budding of COPII-coated carriers from the ER. Based on its ability to bind and bend membranes, Sar1 is a logical candidate for acting as a curvature-sensitive module within the COPII complex. To test this idea, we used two independent approaches to explore the ability of Sar1 isoforms to discriminate between membranes of varying curvatures. First, we compared the binding affinities of SAR-1 for liposomes ranging in size from 105 to 225 nm in diameter, using a co-sedimentation assay. Chemically defined liposomes composed of 66% DOPC, 21% DOPE, 8% DOPS, 5% DOPA, and supplemented with 20% cholesterol, were utilized for these experiments as described previously (13). Because this assay was carried out under equilibrium conditions, we loaded SAR-1 (4 μm) with the poorly hydrolysable GTP analog GMP-PNP to ensure that it remained in an active state throughout the experiment. To monitor binding, we mixed SAR-1 with different concentrations of liposomes and tracked depletion of the protein from the supernatants after centrifugation. The resulting titration curves indicated a 2-fold difference in the apparent dissociation constants between the smallest and largest liposomes, suggesting that SAR-1 prefers membranes of higher curvature (Fig. 3, *A* and *B*).

Because Sar1 is known to remodel membranes, which may influence the binding affinities determined following the prolonged incubation periods needed for sedimentation assays, we decided to use a second approach that measures the association between proteins and membranes instantaneously. Specifically, a rapid mixing fluorescence resonance energy transfer (FRET)-based assay was used to monitor the association of Sar1 with liposomes ranging in average size from 105 to 225 nm in diameter. Importantly, we took advantage of two native tryptophan residues within the *C. elegans* SAR-1 amino terminus, which could act as FRET donors for headgroup-dansylated phosphatidylethanolamine (PE) that was incorporated into liposomes. Using stopped-flow spectroscopy, GTP-loaded SAR-1 (8 μm) was rapidly mixed with liposomes of varying diameters, enabling us to calculate relative binding affinities. Our data demonstrated again that SAR-1 binds preferentially to more highly curved membranes, exhibiting an ∼4.6-fold higher affinity for 105-nm liposomes as compared with 225-nm liposomes (Fig. 3, *C–E*). The difference in apparent binding affinities determined using the two methods likely reflects the ability of SAR-1 to tubulate the larger liposomes over time, thereby increasing its affinity for the 225-nm liposomes during the 1-h co-sedimentation process. Taken together, these data strongly suggest that Sar1 binds to membranes in a curvature-sensitive manner.

##### Sar1 Remodels Supported Lipid Bilayers in a Manner Dependent on GTP Hydrolysis

Current approaches to study the role of Sar1 in membrane remodeling include the use of COPII budding assays in semi-intact cells followed by differential centrifugation or imaging of synthetic liposomes and GUVs by light and electron microscopy (14–16, 26, 35). In cell-free assays, contradictory findings argue both for and against the necessity of Sar1 GTP hydrolysis during cargo secretion from ER membranes (14, 26, 35). In some reports, poorly hydrolysable analogs of GTP potently inhibit the formation of COPII transport carriers, whereas other studies have found that these analogs work equivalently to GTP (14, 26, 35). An explanation for these discrepancies remains unclear but may depend on sample handling. Nevertheless, these data suggest that cell-free assays for COPII budding exhibit substantial variability and are therefore difficult to interpret. EM-based studies using recombinant Sar1 and chemically defined liposomes or GUVs have yielded similarly conflicting results regarding the importance of GTP hydrolysis in membrane scission (7, 15, 18). Again, EM methods employed may be highly sensitive to sample manipulation. Although live imaging assays cause the least perturbation, the resolution afforded by this approach is typically limited and has only demonstrated that Sar1 tubulates membranes in the presence of GTP (16).

**FIGURE 4.**
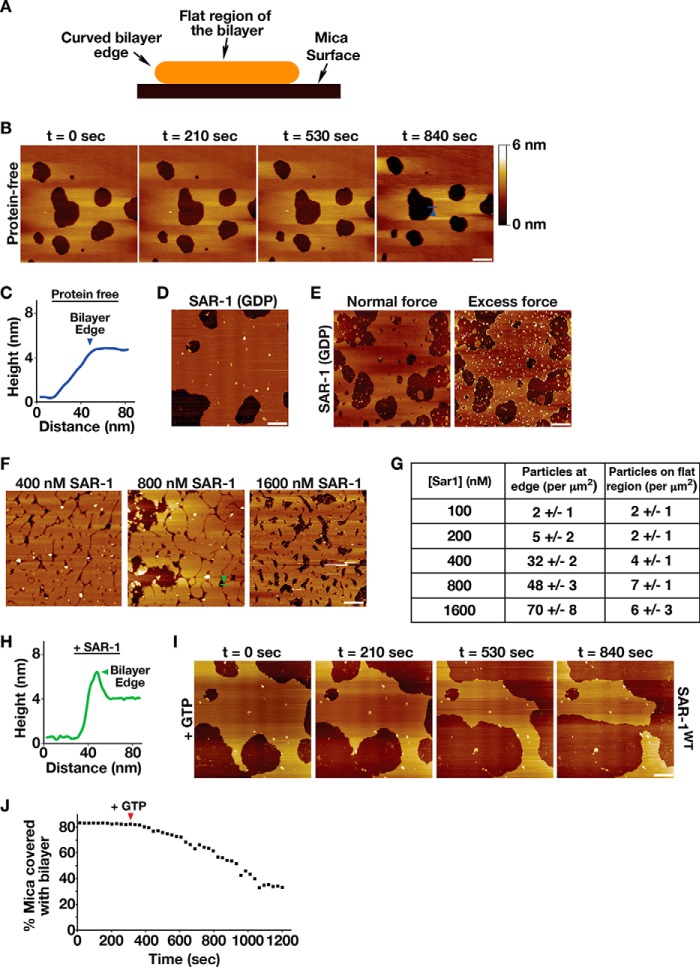
**GTP hydrolysis is necessary for Sar1-mediated membrane remodeling.** *A,* schematic illustrating a lipid bilayer (*orange*) assembled on a mica surface (*black*). The central portion of the bilayer is flat (∼4–5 nm in thickness), and the edges of the bilayer are highly curved. *B,* representative AFM images of supported lipid bilayers (composed of the basic mix of lipids) acquired over time, following assembly in the absence of protein. A *shade-height bar* is shown on the *right. Bar*, 250 nm. *C,* height relative to the mica surface is plotted along the line shown in *B* (*right panel,* highlighted in *blue*). The position of the *arrowhead* (at the bilayer edge) along the line is also shown in *B. D,* representative AFM image of supported lipid bilayer (composed of the basic mix of lipids) assembled in the presence of SAR-1^GDP^ (400 nm). *Bar*, 250 nm. *E,* representative AFM images of the same supported lipid bilayer (composed of the basic mix of lipids), following assembly in the presence of SAR-1 (400 nm), imaged with normal (*left*) or excessive (*right*) force by the AFM tip. *Bar,* 250 nm. *F,* representative AFM images of supported lipid bilayers (composed of the basic mix of lipids) generated in the presence of different concentrations of SAR-1. *Bar,* 250 nm. *G, table* showing the number of particles (per μm^2^) present at the bilayer edge or the flat portion of the bilayer generated in the presence of varying concentrations of SAR-1. *H,* height relative to the mica surface is plotted along the line shown in *F* (*right panel,* highlighted in *green*). The position of the *arrowhead* (at the bilayer edge) along the line is also shown in *F*, which is raised above the height of the bilayer surface suggesting the presence of protein. *I,* representative AFM images of supported lipid bilayers (composed of the basic mix of lipids) imaged over time, following assembly in the presence of SAR-1^GDP^ (400 nm) and supplementation with a buffer containing 1 mm GTP. The times shown are relative to the timing of GTP addition. *Bar,* 250 nm. *J,* representative plot of the area of the mica surface coated with membrane (assembled in the presence of SAR-1^GDP^) over time. The timing of GTP addition is shown with an *arrowhead*.

To study the action of Sar1 on lipid bilayers, we took an alternative approach that uses AFM imaging under fluid. In this method, Sar1 assembly was monitored at nanometer resolution on supported lipid bilayers (SLBs) assembled on mica, with very limited mechanical perturbation. We used SLBs composed of either a basic mix of lipids (70% phosphatidylcholine, 15% PE, and 15% phosphatidylserine) or the major/minor mix (52% DOPC, 21% DOPE, 8% DOPS, 5% DOPA, 9% PI, 2.2% PI4P, 0.8% PI(4,5)P_2_, 2% DAG), which was shown previously to be important for maximal Sar1 membrane binding and facilitating Sec23-Sec24-mediated GAP activity on Sar1 (13). Gaps in the bilayer that formed spontaneously, irrespective of protein addition or lipid composition, produced edges with high curvature (Fig. 4*A*), enabling us to determine the distribution of Sar1 in the presence of both flat and highly curved membrane surfaces. We previously used a similar AFM-based assay to study the formation and activity of the ESCRT-III complex, another protein machinery implicated in membrane bending and scission, and demonstrated that it assembled in a curvature-dependent manner and promoted remodeling of the bent bilayer edges (36). Importantly, by imaging in a fast-scan mode, we were able to visualize the effects of various nucleotides, nucleotide analogs, and other proteins on Sar1 membrane association and remodeling.

Supported lipid bilayers composed of either the basic mix or the major/minor mix formed in the absence of proteins exhibited mild movements on the mica surface, indicating that they remained mobile throughout the period of AFM imaging (Fig. 4*B* and supplemental Video 1). Cross-sections showed that the height of the bilayer was largely uniform across flat regions but exhibited a steep decline at its edges where the mica was exposed (Fig. 4*C*). When supplemented with SAR-1 (400 nm) that had been purified in the presence of GDP, we observed the appearance of particles, which were distributed across the bilayer and at the periphery of the gaps that formed during bilayer assembly (Fig. 4*D*). Decoration of the bilayer edges was particularly evident when excessive force was applied onto the bilayer with the AFM tip (Fig. 4*E*). To verify that these particles corresponded to SAR-1, we varied the concentration of the protein used (100 nm to 1.6 μm) and measured the number of particles observed by AFM. Our findings showed that increased levels of SAR-1 led to the appearance of more particles on the bilayer, which accumulated predominantly at the highly bent edges of the membranes (Fig. 4, *F–H*). Together, these data highlight our ability to monitor SAR-1 accumulation on supported lipid bilayers using AFM.

To facilitate nucleotide exchange onto SAR-1 in the absence of its GEF, a buffer containing 1 mm GTP (equivalent to ∼2-fold the concentration found in most mammalian cell types) was flowed onto a bilayer composed of the basic mix of lipids. Within moments, the bilayer began to undergo a remodeling process that resulted in its systematic removal from the mica surface, which we quantified by measuring the percentage of the mica surface covered with membrane over time (Fig. 4, *I* and *J,* and supplemental Video 2). Based on time-lapse AFM imaging, membrane removal appeared to occur from the highly curved edges of the bilayer (Fig. 4*I*), consistent with our data indicating that SAR-1 can sense membrane curvature. In contrast to the effect of SAR-1 at bent membranes, its presence on the flat regions of the bilayer failed to affect membrane topology under these conditions. The addition of GTP to protein-free bilayers had no substantial effect on their architecture as compared with buffer alone, and the addition of GDP to bilayers harboring SAR-1 similarly had no effect (supplemental Videos 3 and 4). Notably, bilayer removal mediated by SAR-1 was distinct from that of a detergent, which caused the membrane to dissociate from the mica surface in a manner that did not depend on membrane curvature (supplemental Video 5). These data support the idea that GTP-bound SAR-1 is capable of remodeling curved lipid bilayers.

**FIGURE 5.**
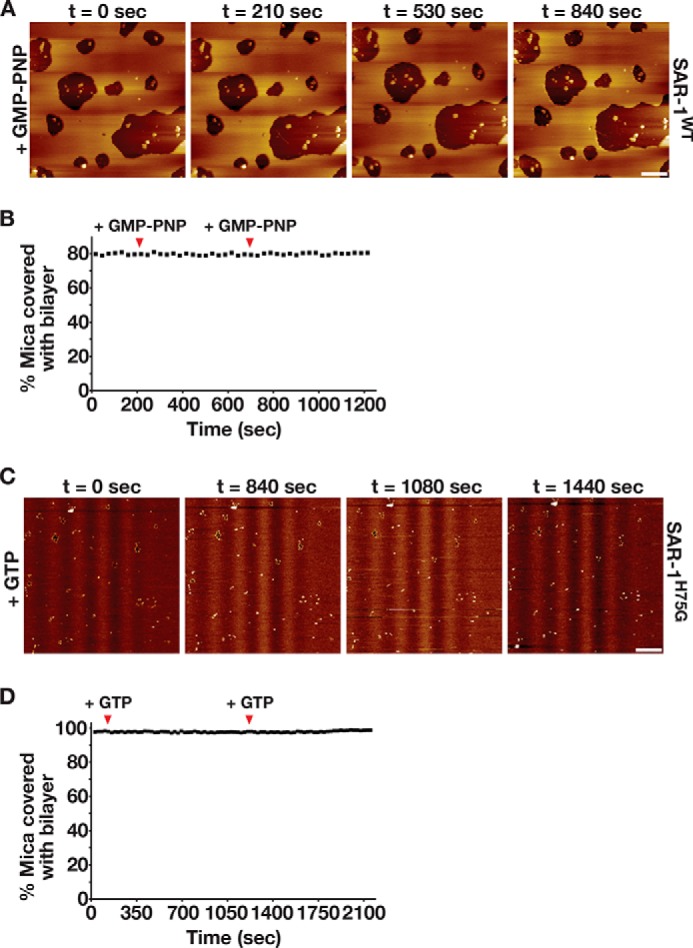
**In the absence of GTP hydrolysis, SAR-1 is unable to remodel membranes.** *A,* representative AFM images of supported lipid bilayers (composed of the basic mix of lipids) imaged over time, following assembly in the presence of SAR-1^GDP^ (1 μm) and supplementation with a buffer containing 1 mm GMP-PNP. *Bar,* 250 nm. *B,* representative plot of the area of the mica surface coated with membrane (assembled in the presence of 1 μm SAR-1^GDP^) over time. The time points of GMP-PNP addition are highlighted with *arrowheads. C,* representative AFM images of supported lipid bilayers (composed of the basic mix of lipids) imaged over time, following assembly in the presence of SAR-1^H75G^ (1 μm) and repeated supplementation with a buffer containing 1 mm GTP. *Bar,* 250 nm. *D,* representative plot of the area of the mica surface coated with membrane (assembled in the presence of 1 μm SAR-1^H75G^) over time. The timepoints of GTP addition are highlighted with *arrowheads*.

To address the question of whether GTP hydrolysis is necessary for membrane remodeling and removal mediated by Sar1, SLBs composed of the basic mix and harboring SAR-1^GDP^ (400 nm) were exposed to an excess of GMP-PNP (1 mm). In contrast to the effect of GTP, we observed only a transient change in bilayer appearance, which was arrested within moments after addition of the nucleotide analog (Fig. 5, *A* and *B*, and supplemental Video 6). We also examined the ability of a mutant form of Sar1 (H75G), which harbors a point mutation within its catalytic switch II region and renders the protein incapable of GTP hydrolysis at any concentration (37), to act on supported lipid bilayers. Although we found SAR-1^H75G^ was highly enriched along membrane edges, the addition of GTP (1 mm) did not stimulate membrane removal, even upon repeated exposure (Fig. 5, *C* and *D*, and supplemental Video 7). Together, these data argue that GTP hydrolysis is necessary for the continuous function of SAR-1 to remodel and sever lipid bilayers.

**FIGURE 6.**
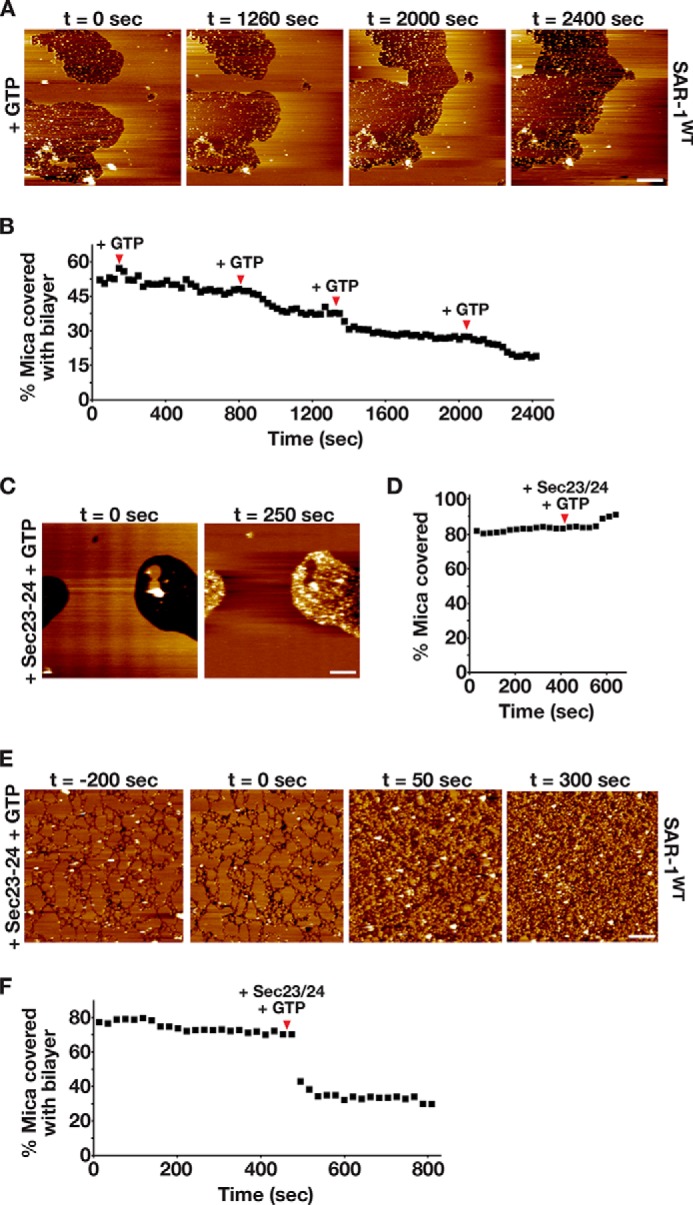
**GAP activity on SAR-1 dramatically accelerates the kinetics of lipid bilayer remodeling.** *A,* representative AFM images of supported lipid bilayers (composed of the minor/minor mix of lipids) imaged over time, following assembly in the presence of SAR-1^GDP^ (400 nm) and supplementation with a buffer containing 1 mm GTP. *Bar,* 250 nm. *B,* representative plot of the area of the mica surface coated with membrane (assembled in the presence of 400 nm SAR-1^GDP^) over time. The time points of GTP addition are highlighted with *arrowheads. C,* representative AFM images of supported lipid bilayers (composed of the major/minor mix of lipids) imaged before and after the addition of purified Sec23-Sec24 (500 nm) and GTP (1 mm). *Bar,* 250 nm. *D,* representative plot of the area of the mica surface coated with membrane and protein (assembled in the presence of 400 nm SAR-1^GDP^) over time. The time point of Sec23-Sec24 and GTP addition is highlighted with an *arrowhead. E,* representative AFM images of supported lipid bilayers (composed of the major/minor mix of lipids) imaged over time, following assembly in the presence of 400 nm SAR-1^GDP^ and supplementation with a buffer containing 1 mm GTP and 500 nm Sec23-Sec24 (at the 0-s time point). *Bar,* 250 nm. *F,* representative plot of the area of the mica surface coated with membrane and protein (assembled in the presence of 400 nm SAR-1^GDP^) over time. The time point of GTP and Sec23-Sec24 addition is highlighted with an *arrowhead*.

To further confirm the importance of GTP hydrolysis for SAR-1-mediated membrane remodeling, we also examined the effect of adding *C. elegans* Sec23-Sec24 (500 nm), the SAR-1 GAP, together with GTP (1 mm) to bilayers. For these studies, we used membranes composed of the major/minor mix, which was shown previously to be necessary for efficient Sec23-Sec24-mediated GAP activity on Sar1 (13). Similar to our findings using bilayers composed of the basic mix, the addition of GTP alone to the major/minor mix membranes containing SAR-1 initiated its steady removal (Fig. 6, *A* and *B*, and supplemental Video 8), although the kinetics of the process was slower and multiple additions of GTP were required. The addition of Sec23-Sec24 and GTP to bilayers lacking SAR-1 had no effect on the membrane (Fig. 6, *C* and *D*, and supplemental Video 9), consistent with the inability of Sec23-Sec24 to bind lipid bilayers without SAR-1 (13). However, we observed substantial, nonspecific accumulation of Sec23-Sec24 heterodimers on the mica surface (Fig. 6, *C* and *D*, and supplemental Video 9). We next added Sec23-Sec24 and GTP to major/minor mix bilayers containing SAR-1 and observed an extremely rapid removal of the membrane (Fig. 6, *E* and *F*, and supplemental Video 10). These data are again consistent with the idea that GTP hydrolysis on SAR-1, which is stimulated by Sec23-Sec24, is critical for promoting membrane remodeling by SAR-1.

##### Membrane Curvature Regulates Sar1 GTP Hydrolysis

**FIGURE 7.**
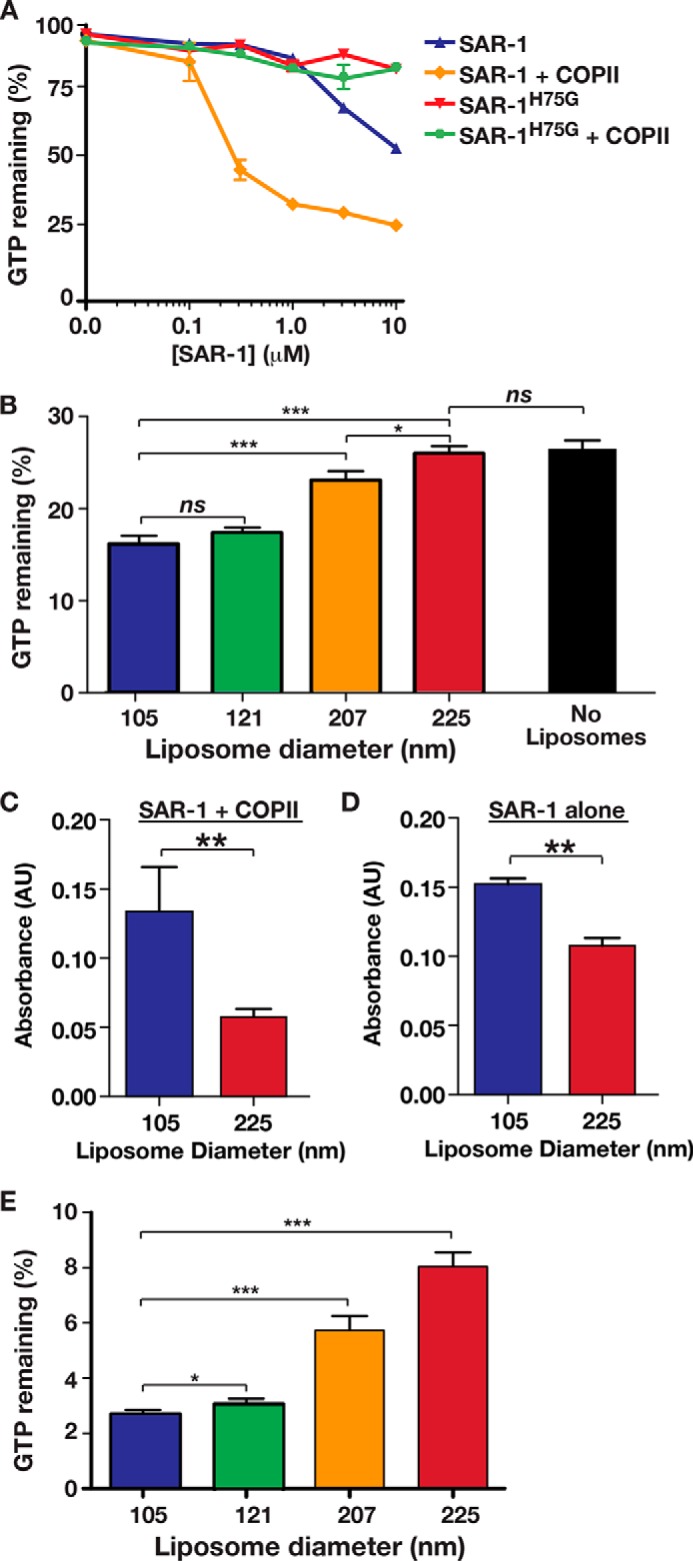
**Membrane curvature regulates GTP hydrolysis on Sar1.** *A,* GTP hydrolysis on SAR-1 was measured using a bioluminescence assay, in the presence or absence of additional COPII subunits and GTP. *B,* GTP hydrolysis on *C. elegans* SAR-1 (3.1 μm) was measured using a bioluminescence assay in the presence of the inner COPII coat components (255 nm) and liposomes composed of the major/minor mix lipids (1 mm) of varying diameter. For comparison, GTP hydrolysis on SAR-1 (3.1 μm) was also measured in the presence of inner COPII coat subunits (255 nm) but in the absence of liposomes (*right bar*). The statistical significance of pairwise differences was calculated using a *t* test. *ns*, not significant; *, *p* < 0.05; ***, *p* < 0.001. *C,* GTP hydrolysis on SAR-1 (14 μm) was measured in the presence of two differently sized liposomes composed of the major/minor mix lipids (1.27 mm) and Sec23-Sec24 (10 μm) using a Malachite Green phosphate assay. The statistical significance of the pairwise difference was calculated using a *t* test. **, *p* < 0.01. *D,* GTP hydrolysis on SAR-1 (14 μm) was measured in the presence of two differently sized liposomes composed of the major/minor mix lipids (1.27 mm) using a Malachite Green phosphate assay. The statistical significance of the pairwise difference was calculated using a *t* test. **, *p* < 0.01. *E,* GTP hydrolysis on *S. cerevisiae* Sar1p (3.1 μm) was measured using a bioluminescent assay in the presence of the inner COPII coat components (255 nm) and liposomes composed of the major/minor mix lipids (1 mm) of varying diameter. The statistical significance of pairwise differences was calculated using a *t* test. *ns*, not significant; *, *p* < 0.05; ***, *p* < 0.001.

Given the ability of SAR-1 to bind preferentially to membranes of high curvature and remodel them in a manner that depends on GTP hydrolysis, we questioned whether curvature may also influence the rate of GTP hydrolysis on Sar1. To examine this possibility, we employed a new, facile, and highly reproducible bioluminescence assay for monitoring the enzymatic activity of Sar1 in the presence of liposomes that ranged in average size from 105 to 225 nm in diameter. To first validate the use of this assay, we measured Sar1-mediated GTP hydrolysis over a range of protein concentrations. These data highlight the ability of SAR-1 to hydrolyze GTP in solution, but only at relatively high concentrations (above 4 μm) (Fig. 7*A*). The mutant form of SAR-1 (H75G) was largely incapable of GTP hydrolysis at any concentration, as predicted by previous work (Fig. 7*A*). Additionally, the supplementation of the reaction with recombinant Sec23-Sec24 dramatically increased the rate of GTP hydrolysis by wild type SAR-1 but not SAR-1^H75G^ (Fig. 7*A*). These findings collectively indicate that the bioluminescent assay provides a faithful readout of Sar1 GTPase activity.

To test whether membrane curvature affects Sar1 GTPase activity, we incubated nucleotide-free SAR-1 (3.1 μm) with Sec23-Sec24 heterodimers (255 nm) and liposomes (1 mm) of various curvatures and initiated each reaction by the addition of 5 μm GTP. Based on our sedimentation assays, equivalent amounts of SAR-1 are bound to each liposome population under these conditions, irrespective of their diameter (Fig. 3, *A* and *B*). Our studies indicated that GTPase activity was inversely related to liposome size, with the 105-nm liposomes promoting SAR-1 activity significantly better than liposomes greater than 200 nm in diameter (Fig. 7*B*). Additionally, by comparing these data to GTPase activity measured in the absence of membranes, we found that the largest liposomes used (225 nm in diameter) failed to significantly stimulate GTP hydrolysis on SAR-1, in contrast to the smaller liposomes (Fig. 7*B*). We also confirmed that SAR-1 exhibits curvature sensitivity in the presence of Sec23-Sec24 using a standard colorimetric assay that measures inorganic phosphate release as a function of GTPase activity (Fig. 7*C*). To further verify that SAR-1, and not its GAP, exhibits curvature sensitivity, we conducted similar experiments using SAR-1 alone. To detect inorganic phosphate release, a higher concentration of SAR-1 was necessary (14 μm). Under these conditions, we again found that GTP hydrolysis on SAR-1 was increased in the presence of small liposomes (105 nm) as compared with large liposomes (225 nm) (Fig. 7*D*).

To determine whether this curvature-sensitive property of SAR-1 is conserved, we also measured the ability of yeast Sar1p to hydrolyze GTP in the presence of differently sized liposomes. Strikingly, the impact of curvature was even more pronounced, with a significant difference in Sar1p GTPase activity in the presence of 105-nm liposomes as compared with 225-nm liposomes (Fig. 7*E*). These data highlight a new regulatory mechanism that controls Sar1 function and demonstrates the role of membrane curvature in influencing GTP hydrolysis on Sar1. Together, our findings suggest that the various curvatures adopted during individual steps of COPII-mediated transport carrier budding directly influence Sar1 distribution and activity.

## Discussion

The amino-terminal region of Sar1 has been shown to play an essential role in membrane deformation and generating COPII-coated transport carriers, both *in vivo* and *in vitro* (7, 8, 38). However, the mechanisms by which this region binds and manipulates membranes remain unclear. To date, the intact domain has not been crystallographically resolved, but it is predicted to form an amphipathic α-helix and is capable of destabilizing lipid bilayers on its own (39). Models suggest that GTP binding by Sar1 relieves it from an autoinhibited state and enables exposure of its amino terminus to facilitate membrane association (8). In contrast, our data suggest that the amphipathic helix transitions constantly through solvent-exposed states, irrespective of GTP binding. However, GTP loading promotes stable membrane penetration, which is necessary to generate curvature. Thus, our results are consistent with the idea that Sar1 continually samples membranes, but only in the presence of its GEF Sec12 can it stably insert to facilitate the formation of COPII transport carriers.

In addition to its role during the initial stages of COPII carrier formation, Sar1 has also been implicated in membrane sculpting and scission steps (7, 8). Recent work highlighted the propensity of Sar1 to dimerize and form ordered lattices on membrane tubules exhibiting diameters larger than 60 nm, equivalent to the size of the smallest COPII-coated transport carriers observed *in vitro* or *in vivo* (3, 15). These data are consistent with our results demonstrating that Sar1 can form dimers in solution. In cases where membrane tubules fall below 60 nm in diameter, ordered arrays of Sar1 are no longer observed, implying that curvature plays an important role in Sar1 membrane association (15). We explored this idea further by examining the relative binding affinities of Sar1 for a range of membrane curvatures, which COPII transport carriers likely adopt during their formation. Our findings indicate that Sar1 associates more tightly with membranes of elevated curvature. Importantly, discrepancies in the relative affinities obtained using equilibrium binding and stopped-flow rapid kinetics further suggest that Sar1 actively bends the membrane, diminishing lipid density in the outer leaflet as compared with the inner leaflet and facilitating membrane penetration of additional molecules of Sar1. A localized increase in Sar1 concentration is likely instrumental in achieving sufficient bending, potentially through molecular crowding, to ultimately drive membrane scission (39).

In many ways, Sar1 appears to act similarly to epsin, a regulator of clathrin-coated vesicle formation, which harbors an ENTH domain that penetrates the inner leaflet of the plasma membrane to a depth similar to what we have measured for the Sar1 amphipathic helix (40–43). This type of shallow penetration leads to a major asymmetry in the structure of a membrane monolayer, enabling positive curvature generation (44). Although controversial, the epsin ENTH domain has been proposed to have an active role in the vesicle scission process during endocytosis (45, 46). Analogously, by acting as the curvature sensor during COPII transport carrier formation, Sar1 may possess a similar function. Consistent with this idea, at sufficiently high concentrations, Sar1 is capable of transforming membrane tubules into vesicles independently of GTP hydrolysis (15). However, it remains unclear whether the concentration of Sar1 necessary for this effect *in vitro* is physiologically relevant.

Although the current situation is confusing, abundant evidence now exists to support both active and passive roles for Sar1 GTP hydrolysis in generating transport carriers from the ER (1, 7, 8, 13–18). However, fission reactions that occur under conditions where the GTPase cycle is halted typically require high levels of Sar1 to be present, raising concerns. To address this issue, we took advantage of an *in vitro* assay to study the impact of various nucleotides and nucleotide analogs on Sar1-mediated membrane remodeling using a limited concentration of the protein (400 nm). Irrespective of the artificial nature of these experiments, our combined use of supported lipid bilayers and AFM imaging enabled us to visualize the effect of Sar1 under various conditions in real time. Our findings demonstrated that GTP hydrolysis is necessary for membrane remodeling mediated by Sar1, suggesting a requirement to expend energy during this process. These data were particularly surprising in light of our results showing that the intrinsic rate of Sar1 GTP hydrolysis in the absence of its GAP is extremely slow. Nevertheless, this minimal amount of GTP consumption appears to be required for Sar1 to perform work on supported lipid bilayers, as neither GTP-locked Sar1 (H75G) nor wild type Sar1 in the presence of poorly hydrolysable GTP analogs were able to mimic the effect of adding GTP to wild type Sar1 assembled on the edges of membranes.

One possible explanation for these findings stems from our work showing that GTP hydrolysis on Sar1 is enhanced in the presence of membranes with elevated curvature. Although the differences in GTPase activity that we measured may appear modest, we were only able to examine effects over a narrow range of curvatures (∼0.004–0.009 nm^−1^, where curvature is defined as 1/radius). The edges of supported lipid bilayers are predicted to exhibit the highest degree of curvature feasible (∼0.4 nm^−1^), which appears to promote substantial GTP hydrolysis on Sar1, sufficient to remodel the bilayer even in the absence of its GAP. An analogous situation may arise during the fission of COPII-coated transport carriers from the ER. Over the course of this final step, the ER membrane must achieve a high degree of curvature, significantly higher than that of vesicles we can generate by extrusion, which may be sufficient to promote GTP hydrolysis on Sar1 and thereby disrupt the bilayer to enable fission (3). The role of Sar1 GAP activity must also be considered, especially in light of its dramatic enhancement of the kinetics of SAR-1-mediated membrane remodeling. It is feasible that Sec23 behaves similarly to ArfGAP1, the GAP that stimulates GTP hydrolysis on the Arf1 GTPase in a curvature-dependent manner and directs COPI-mediated trafficking (47, 48). However, unlike ArfGAP1, which harbors an ArfGAP1 lipid packing sensor motif that inserts into one leaflet of highly bent bilayers, none of the COPII subunits bind directly to membranes (13). Thus, our data are more consistent with the idea that Sar1, and not its GAP, acts as the curvature sensor in COPII-mediated membrane transport.

Taken together, our findings support a model in which Sec12-mediated GTP loading onto Sar1 allows for stable membrane penetration of its amphipathic tail to initiate membrane curvature on subdomains of the ER and recruitment of additional coat components. A number of regulatory factors, including Sec16, likely contribute to the control of Sec23-dependent GAP activity to limit release of Sar1 from the membrane (49, 50). Ultimately, we speculate that the progressive ability of Sar1 to bend membranes, likely in coordination with COPII coat assembly, leads to an increased propensity for GTP hydrolysis, which is able to support membrane fission and the release of coated transport carriers.

## Author Contributions

M. G. H., I. M., R. M. H., E. R. C., J. M. E., and A. A. conceived and designed the experiments. M. G. H., I. M., L. W., E. R. C., J. M. E., and A. A. performed the experiments and analyzed the data. M. G. H. and A. A. wrote the paper.

## Footnotes

4 The abbreviations used are:

ER: endoplasmic reticulum
DOPC: 1,2-dioleoyl-*sn*-glycero-3-phosphocholine
DOPE: 1,2-dioleoyl-*sn*-glycero-3-phosphoethanolamine
DOPS: 1,2-dioleoyl-*sn*-glycero-3-phosphoserine
DOPA: 1,2-dioleoyl-*sn*-glycero-3-phosphate
PI: phosphatidylinositol
PI4P: phosphatidylinositol 4-phosphate
PI4,5P_2_: phosphatidylinositol 4,5-bisphosphate
DAG: diacylglycerol
NBD-PE: 1,2-dioleoyl-*sn*-glycero-3-phosphoethanolamine-*N*-(7-nitro-2–1,3-benzoxadiazol-4-yl)
GEF: guanine nucleotideexchange factor
GUV: giant unilamellar vesicle
AFM: atomic force microscopy
SLB: supported lipid bilayer
COPII: coat protein II
MALS: multiangle light scattering
GMP-PNP: guanosine-5′-[β,γ-imido]triphosphate
dansyl: 5-dimethylaminonaphthalene-1-sulfonyl
GAP: guanine nucleotide-activating protein
PE: phosphatidylethanolamine
6,7-Br_2_-PC: 1-palmitoyl-2-stearoyl-(6,7)-dibromo-*sn*-glycero-3-phosphocholine
11,12-Br_2_-PC: 1-palmitoyl-2-stearoyl-(11,12)-dibromo-*sn*-glycero-3-phosphocholine.
